# A genomic perspective of the pink-headed duck *Rhodonessa caryophyllacea* suggests a long history of low effective population size

**DOI:** 10.1038/s41598-017-16975-1

**Published:** 2017-12-04

**Authors:** Per G. P. Ericson, Yanhua Qu, Mozes P. K. Blom, Ulf S. Johansson, Martin Irestedt

**Affiliations:** 1 Department of Bioinformatics and Genetics, Swedish Museum of Natural History, PO Box 50007, Stockholm, SE-10405 Sweden; 2 Key Laboratory of Zoological Systematics and Evolution, Institute of Zoology, Chinese Academy of Sciences, Beijing, 100101 China; 3 Department of Zoology, Swedish Museum of Natural History, PO Box 50007, Stockholm, SE-10405 Sweden

## Abstract

The first molecular phylogenetic hypothesis for the possibly extinct pink-headed duck *Rhodonessa caryophyllacea* unambiguously shows that it belongs to the pochard radiation that also includes the genera *Aythya* and *Netta*. It is the sister to all modern-day pochards and belongs to a lineage that branched off from the others more than 2.8 million years ago. *Rhodonessa caryophyllacea* is believed to never have been common in modern time and we show this has probably been the situation for as long as 100,000 years. Our results suggest that their effective population size varied between 15,000 and 25,000 individuals during the last 150,000 years of the Pleistocene. The reasons behind this are largely unknown as very little is known about the life-history and biology of this species. Presumably it is due to factors related to feeding or to breeding, but we may never know this for sure.

## Introduction

The pink-headed duck (*Rhodonessa caryophyllacea*) is one of the rarest and most sought after bird species in Asia. The pink head and neck in males gives it an appearance as no other duck. The colour on these pink feathers is formed by carotenoid-pigments, which is a near-unique trait as carotenoid-pigmented feathers among waterfowl species (Anseriformes) only has been reported from one additional duck species^1^.

*Rhodonessa caryophyllacea* was once locally distributed in the wetlands of India, Bangladesh and Myanmar, and occurred rarely in Nepal, with most records from north-east India and adjacent Bangladesh^2^. It is supposed to have been uncommon and rare even before the population began to decline due to hunting and habitat loss. The last definite record in the wild was in 1949. After this there are a couple of less certain sightings from Myanmar between 2003 and 2006. Several directed surveys in Myanmar in recent years have not been successful in finding the species. For the time being *Rhodonessa caryophyllacea* is placed in the category “critically endangered” by IUCN^2^.

When originally described by John Latham in 1790 the pink-headed duck was placed in the genus *Anas*. Later Reichenbach^3^ erected the genus *Rhodonessa* of which he considered the pink-headed duck to be the only member. The family Anatidae (ducks, geese, swans and their allies) are divided into several tribes based on a variety of morphological and behavioural characters^4^. *R. caryophyllacea* has variably been considered as belonging to either the tribe Anatini (dabbling ducks) or Aythyini (pochards), depending on what kind of information has been regarded as taxonomically most reliable. While Delacour and Mayr^4^ considered *R. caryophyllacea* as an aberrant member of the dabbling ducks, others have referred to a number of morphological characters that place it closer to the pochards^5–9^. Humphrey and Ripley^7^ writes: “*Rhodonessa* has had an uncertain status because it combines some of the characters of two very different groups of waterfowl: the pochards on the one hand, and on the other, ducks which are better adapted for a more terrestrial existence, namely the dabbling ducks and perching ducks. This combination of characters has led some workers to suggest that *Rhodonessa* might be a “link” relating in a phylogenetic sense the pochards and the dabbling ducks”.

Livezey^8^, in an analysis of 120 morphological characters, found a most parsimonious tree in which *Rhodonessa* grouped together with the genera *Netta* and *Aythya* in a clade that was sister to *Marmaronetta*, a phylogenetic placement based on seven skeletal synapomorphies. Furthermore, *Netta* and *Aythya* grouped together, on the exclusion of *R. caryophyllacea*, based on a single character of the tarsometatarsus (the anterior extent of the internal and external ridges of the shaft are essentially equal in *R*. *caryophyllacea*, while the internal ridge extends less prominent anteriorly than the external in *Netta* and *Aythya*). In a subsequent study focusing on the phylogeny of the subfamily Aythyini, Livezey^9^ instead found *R. caryophyllacea* to be sister to the red-crested pochard (*Netta rufina*) and suggested the two species to be placed in the genus *Rhodonessa* (this name has priority according to the International Code of Zoological Nomenclature). This taxonomic treatment has not been followed by subsequent authors, however^10^. state that *caryophyllacea* should be placed in an own genus based on the many unique characteristics that separate it from all other Aythini, including *Netta*.

In this paper we present the first molecular analysis of the phylogenetic position of *Rhodonessa caryophyllacea*. These results are compared with previous phylogenetic hypotheses based on morphological data. We also present a tentative analysis of this species’ population history during the last 100,000 years utilizing genomic data.

## Results and Discussion

Contrary to what sometimes has been suggested (e.g.^4^), the phylogenetic analysis confirms that *Rhodonessa caryophyllacea* is closer to the pochards (Aythyini) than to the dabbling-ducks (Anatini) (Supplementary Fig. S1). It forms, together with *Netta rufina* and *N. peposaca* and all *Aythya* species, the sister clade to *Asarcornis scutulata* and *Marmaronetta angustirostris*. Within this clade there is strong support for *R. caryophyllacea* being sister to all the other species. The average mitochondrial genetic distance between *R. caryophyllacea* and the eight representative species of *Aythya* and *Netta* is 6.5 percent (s.d. = 0.35, uncorrected p-value). Applying an average mitochondrial mutation rate of 2.29 percent per site per million years^11^ it is hypothesized that the ancestor of *R. caryophyllacea* was separated from the ancestor of the other species in this clade 2.8 million years ago.

In this first molecular phylogenetic hypothesis, the position of *R. caryophyllacea* differs from all previously suggested hypotheses based on morphology. For example, Livezey^9^ placed *R. caryophyllacea* as sister to *N. rufina*, a clade that in turn was sister with the other species of *Netta* (*peposaca* and *erythrophthalma*). Should the molecular phylogeny be correct all the five morphological characters (one skeletal and four plumage coloration patterns) proposed by Livezey^9^ in support of a *R. caryophyllacea* - *N. rufina* clade are either misinterpreted, symplesiomorphic or convergently evolved. The same is true for the four plumage characters that were suggested to be synapomorphies of a *R. caryophyllacea* - *Netta* clade to the exclusion of all *Aythya*-species^9^.

**Figure 1:**
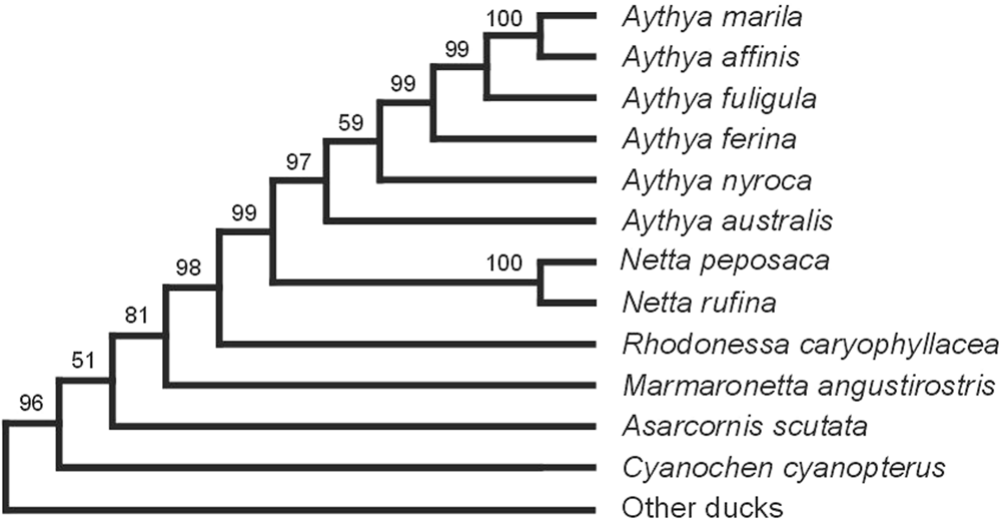
The maximum-likelihood tree resulting from the analysis of a data set consisting of 2086 bp (cytochrome *b* and NADH dehydrogenase subunit 2 genes) obtained from 66 species of ducks, geese and swans. Here is shown the part of the tree that includes the genera *Aythya* and *Netta* (tribe Aythyini) together with the species that fell immediately outside that clade, including *Rhodonessa caryophyllacea*. Numbers at the nodes are bootstrap values (after 100 replicates).The full maximum-likelihood tree is shown in the Supplementary material.

When mapping Livezey’s^9^ morphological characters on the pochard-portion of the tree derived in the molecular analysis (Fig. 1) the number of steps (i.e. character transformations) is 122. When the characters’ distributions are resolved in the most parsimonious way (tree in Fig. 1 in Livezey 1996) the number of steps is 130. Interestingly when Livezey’s characters are mapped on the molecular tree there is a strong support by unambiguous character transformations (i.e. no convergences or reversals) for the nodes of interest herein (Supplementary Table S1). There are four synapomorhies (one skeletal, two natal and one definite integument) for the clade with the genera *Marmaronetta*, *Rhodonessa*, *Netta* and *Aythya*, no less than thirteen synapomorphies for the clade consisting of *Rhodonessa*, *Netta* and *Aythya* (six skeletal, two trachea and five definite integument), and three synapomorphies for the clade with *Netta* and *Aythya* (one skeletal, one trachea and one definite integument). Finally, a monophyletic *Aythya* clade is supported by five unambiguous character transformations (four skeletal and one definite integument). It is evident that morphology strongly supports the topology of this part of the molecular tree. Still, when all morphological characters are taken together and their distributions mapped across all seventeen species of ducks included in Livezey’s^9^ analysis, the most parsimonious distribution favours a different topology for the tree with the pochards and their closest allies. If we assume that the molecular tree reflects the true evolutionary species tree better than the morphological tree it becomes clear that some homology assessments of the morphological characters must be incorrect. The character states may not be homologous or are subject to homoplasy (convergences and/or reversals). Parsimony arguably is the most reasonable criterion to apply when choosing between many alternate ways to resolve character distributions among taxa. Still, the critical problem faced by students of morphology is the limited understanding of the genetic foundations of most phenotypic traits. Homology assessments are often made based on subjective opinions about what is regarded as a true, inherited similarity and what may have developed independently. That this is a real problem is shown by the observation that the average consistency index for individual, morphological characters across a most parsimonious tree often approaches 0.50 in published studies (in Livezey 1996 it is 0.68). If the vast majority of characters were correctly interpreted one would expect the average index to approach 1.0. One may argue that once a tree that is thought to reflect the true evolutionary relationships for the group has been chosen (based on the parsimony criterion or in another way), then all character distributions that do not fit per definition must be wrongly interpreted and should thus be excluded from the analysis as they otherwise risk to compromising the analysis. One should then re-analyse the data and study the character distributions again. This approach, called “reciprocal illumination”^12^, has been criticised for approaching circularity and has in reality rarely been used. An approach that could prove more promising is to map the morphological characters on a well-supported tree derived from molecular data to find what characters are in need for re-study and re-interpretation. It is likely one would find that certain suits of characters are less susceptible to convergences than others providing a mean to focus the morphological studies on such characters. In the molecular era such an increased knowledge would perhaps be most valuable to palaeontologists who often have to rely entirely on the interpretations of the morphology of their study organisms.

**Figure 2:**
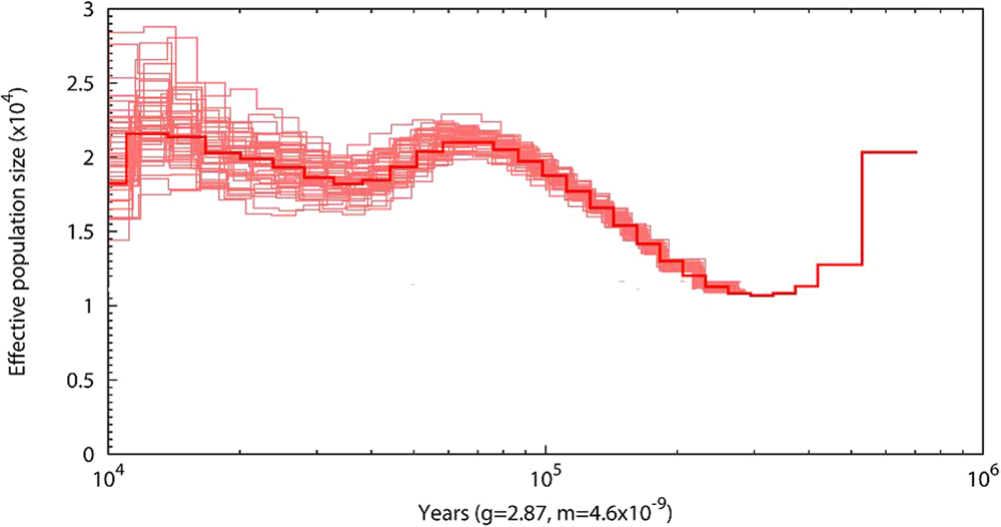
The change in effective population size over time for *Rhodonessa caryophyllacea* was derived by the pairwise sequential Markovian coalescent model (PSMC). The x axis gives a log scale of the time in years, applying a genome mutation rate of 4.6 * 10-9 per site and generation time of 2.86 years. The bold red line shows the effective population size through time. The thin pink lines represent 50 rounds of bootstrapped sequences.

The information on the demographic history provided by the PSMC analysis should be interpreted with some caution as the amount of sequence data available does not fulfil recommendations for what is needed to make reliable inference of population size dynamics over time. Nadachowska-Brzyska *et al*.^13^ found that one should strive for a sequencing depth of 18X or above. For the *Rhodonessa caryophyllacea* genome we have obtained only a 7X coverage. Although the precision of the results will increase with more data we believe that some general observations from the present study (Fig. 2) will hold up. For example, although the effective population size of *R. caryophyllacea* has fluctuated over time it seems to have always been quite small, maybe less than 25,000 individuals. The PSMC analysis does not provide information on the last 10,000 years but it seems likely that the population has remained low in numbers in the Holocene. This would have made the species particularly vulnerable to the negative factors imposed by humans in terms of hunting and habitat changes following the growth and expansion of the human population, converting lowland wetlands to cultivation.

## Material and Methods

**Figure 3:**
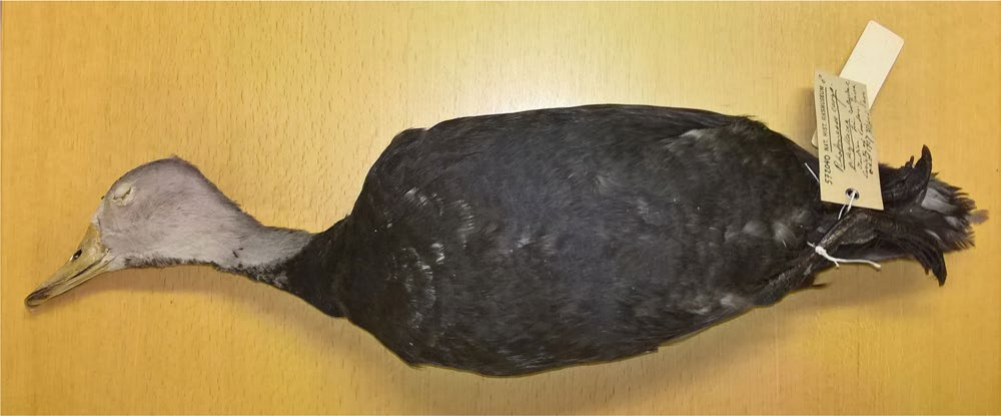
Skin of adult male of Pink-headed Duck (*Rhodonessa caryophyllacea*) kept at the Swedish Museum of Natural History (NRM 572040). The bird was captured wild in “India” (no more detailed information given) and brought to England where it died in 1927.

DNA was extracted from a foot-pad sample from a mounted skin of *Rhodonessa caryophyllacea* (NRM 572040, Fig. 3) kept at the Swedish Museum of Natural History. This is an adult male that was captured wild in “India” (no more detailed information given) and brought to England where it died in 1927. The extraction protocol follows Irestedt *et al*.^14^. The DNA in such an old specimen typically holds quite degraded DNA. Still, DNA obtained from museum study skins have been used in science since the late 1980’s (e.g.^14–16^, mostly by the use of traditional amplicon-based Sanger-sequencing. More recently next-generation sequencing technologies have made it possible to sequence millions of short DNA-fragments. Compared to Sanger-sequencing the new techniques are less time consuming and considerably cheaper. The NGS techniques opens up for genetic studies of the millions of animals and plants stored in museums worldwide making “museomics” a rising field in biology (e.g.^17^). It is now possible to evaluate the evolutionary and biogeographic history for species for which museum specimens were collected many decades ago and for which modern day samples are hard to obtain or are no longer available due to recent extinction. *R. caryophyllacea* is definitely an example of such a species.

Most museum specimens of *R. caryophyllacea* are preserved as mounted birds or skins. Very few anatomical specimens exist in the world; three partial and three complete skeletons are kept in Natural History Museum in London, Royal Museum of Belgium, and Field Museum of Natural History, Chicago^9,18^, and all three known alcohol specimens are kept in the Natural History Museum, London^19^. Although no fresh tissue suitable for genetic analysis exists for this rare species, good quality, genome-wide resequence data can still be obtained from old museum specimens using NGS techniques.

### Library preparation, sequencing, assembly and bioinformatics

The preparation of paired-end genome libraries for Illumina high-throughput sequencing followed the protocol published by Meyer and Kircher^20^), except that no further fragmentation was needed of DNA derived from museum skins as this is already fragmented through natural degradation. The library was pooled with one other species (a woodpecker, *Xiphidiopicus percussus*) and sequenced on a single Illumina Hiseq X lane. The sequencing reads were processed using a custom designed workflow that is available at https://github.com/mozesblom. The workflow removes pcr-duplicates, adapter contamination, low-quality bases, low-complexity reads and merges over-lapping read-pairs. Only reads over 30 bp in length were retained. Both adapter and quality trimming was done using TRIMMOMATIC (v.0.32^21^), pcr duplicates were removed with SuperDeduper (v.1.4^22^), reads were merged with PEAR (v.0.9.6^23^) and overall quality and length distribution of sequence reads were inspected prior and post the clean-up workflow using FASTQC (v.0.11.5^24^).

Since phylogenetic placement remained uncertain and a suitable reference for direct mapping is therefore absent, we assembled the *Rhodonessa caryophyllacea* mitochondrial genome with an iterative baiting and mapping approach (MITObim v.1.8^25^). MITObim finds initial regions of similarity between a target library and a distant reference, and then uses an iterative mapping strategy without use of the original reference to find reads that overlap with these initial segments. This reference free strategy is repeated until the complete mitochondrial genome is assembled or if no further overlapping reads are identified. We randomly subsampled twelve million merged reads using SeqTK (v.1.2^26^) and used the mitochondrial genome of the red-crested pochard *Netta rufina* (NC 024922; Kan and Li unpublished) as initial reference seed. The resulting mitochondrial assembly was subsequently validated, by mapping all sequence reads against the inferred mitogenome using BWA mem^27^. Subsequently, GATK^28^ was used to call any potential variable sites between reads and reference (HaplotypeCaller) and to ultimately generate a consensus sequence (FastaAlternateReferenceMaker)We called genotypes and generated a consensus sequence using GATK (Picard v.2.6).

### Molecular phylogenetics and morphological variation

The phylogenetic position of *Rhodonessa caryophyllacea* within Anatidae was investigated using a data set of mitochondrial genes obtained from Johnson and Sorenson^29^) and Gonzalez *et al*.^30^). After having extracted these genes (cytochrome *b* and NADH dehydrogenase subunit 2) from the *R. caryophyllacea* genome the total data set consisted of 2086 bp from 66 species of ducks, geese and swans. The data set was analyzed by maximum-likelihood using RAxML^31^) applying the General Time Reversible model of nucleotide substitution. The age of the phylogenetic splits between taxa was estimated by applying a divergence rate of 2.29 percent per site per million years for mitochondrial DNA in anseriform birds^11^.

Ninety-nine morphological characters that show variation within the larger clade to which the *Rhodonessa caryophyllacea* and its closest relatives belong were taken from Livezey (1996) (see Supplementary material for the data matrix). We used Mesquite v. 3.2^32^ to analyze the distribution of character-states when mapped onto the morphological tree published by Livezey^9^ and the molecular tree reconstructed herein, respectively.

### Demographic history

A glimpse of the demographic history of the *Rhodonessa caryophyllacea* is obtained by studying the whole genome, for which we used the genome of mallard *Anas platyrhynchos* ^33^ as reference for mapping. The pairwise sequentially Markovian coalescent (PSMC) model uses the coalescent approach to estimate changes in effective population size^34^. Each diploid genome is a collection of hundreds of thousands independent loci. By estimating the time to the most recent common ancestor (TMRCA) of the two alleles at each locus a distribution of TMRCA across the genome is created. Since the rate of coalescent events is inversely proportional to the effective population size^35^, PSMC identifies periods of change in the effective population size. For example, when many loci coalesce at the same time, it is a sign of small effective population size at that particular time.

There are considerable limitations of the PSMC method, however. One is that it cannot recover recent changes in effective population size, i.e. younger than 10,000 years. Another is that it is sensitive to estimates of the generation time and average mutation rate as these are used to scale the TMRCA distribution into years. However, the generation time and mutation rate estimates do not change the shape of the PSMC curve but move the curve along the axes. For example, a halved generation time will double the estimate of the effective population size (given a fixed mutation rate per year), and a halved mutation rate per year will move the curve backward in time and also double the estimate of the effective population size. The general biology and life-history parameters for *R. caryophyllacea* are poorly known. The only generation time given for *R. caryophyllacea* is 7 years (BirdLife International 2016) but the foundation for this is unclear, no reference is given. Furthermore, this seems as a long generation time for a duck. For example, Joseph *et al*.^36^ estimated a generation time of 2 years for two species of dabbling ducks (genus *Anas*). The probably most relevant and reliable data come from two 35-year studies of the pochard species *Aythya fuligula* and *A. ferina*, respectively^37,38^. The estimated generation time in both studies was 2.86 years and this is used here. As estimate of the genomic mutation rate per generation we use 4.6*10-9 that was obtained in a study for the collared flycatcher *Ficedula albicollis* ^39^ and is arguably the most reliable estimate yet for birds. The parameters for the PSMC analysis were set to “ - N30 - t5 - r5 - p 4 + 30*2 + 4 + 6 + 19” following recommendations by Nadachowska-Brzyska^40^. We performed bootstrapping of PSMC by splitting the *R*. *caryophyllacea* genome into shorter segments from which new sequences were constructed through random sampling with replacement.

### Data Availability

GenBank accession numbers MF615511 and MF615512.

## Electronic supplementary material

Supplementary Information
