# Supplementary material for: A genomic perspective of the pink-headed duck Rhodonessa caryophyllacea suggests a long history of low effective population size
Source: Sci Rep. 2017 Dec 4;7:16853. doi: 10.1038/s41598-017-16975-1 (PMC5715134; doi:10.1038/s41598-017-16975-1)

Supplementary information

"A genomic perspective of the Pink-headed Duck *Rhodonessa caryophyllacea* suggests a long history of low effective population size"

Per GP Ericson[1*], Yanhua Qu[1,2], Mozes PK Blom[1], Ulf S Johansson[3] and Martin Irestedt[1]

[1]*Department of Bioinformatics and Genetics, Swedish Museum of Natural History, PO Box 50007, Stockholm 10405, Sweden*

[2] *Key Laboratory of Zoological Systematics and Evolution, Institute of Zoology, Chinese Academy of Sciences, Beijing 100101, China*

[3]*Department of Zoology, Swedish Museum of Natural History, PO Box 50007, Stockholm 10405, Sweden*

* Corresponding author: Per GP Ericson
Email: per.ericson@nrm.se

Supplementary Table S1. Synapomorphies supporting monophyly of four different clades when mapped on to the molecular tree: a) *Marmaronetta*, *Rhodonessa*, *Netta* and *Aythya*, b) *Rhodonessa*, *Netta* and *Aythya*, c) *Netta* and *Aythya*, and d) *Aythya*. Character numbers refer to Livezey (1996). For each character the state observed in the respective clade is written in bold. Note that *N. erythrophtalma* is not considered in this as it was not included in the molecular analysis. Also: *Asarcornis scutulata* is not included in the morphological study.

a) Monophyly of *Marmaronetta, Rhodonessa, Netta* and *Aythya*:

s8 Carpometacarpus, extremitas proximalis carpometacarpi, trochlea carpalis, labrum ventralis, orientation relative to corpus carpometacarpi, facies ventralis: (a) coplanar; **(b) laterally rotated**
n8 Dark preorbital stripe (problematic): (a) present (outgroups); **(b) obsolete or absent** (includes fuligula, marila, and affinis, in which face darkened)
n9 Dark, contrasting auricular spot: (a) present; **(b) absent**.
d44 Metallic-colored speculum: (a) present; **(b) absent**.

b) Monophyly of *Rhodonessa, Netta* and *Aythya*:

s1 Columna vertebralis, vertebrae cervicales, modal number: (a) 16; **(b) 17**.
s3 Sternum, rostrum sterni, labrum interna (character 82): (a) a rounded notch; **(b) rounded notch with small medial point**.
s5 Humerus, extremitas distalis, relative caudal prominence of epicondyla dorsalis and ventralis: (a) epicondyla essentially equal; **(b) epicondylus dorsalis cranial to epicondylus ventralis**.
s6 Carpometacarpus, extremitas proximalis carpometacarpi, trochlea carpalis, labrum dorsalis, rounded prominence on distal terminus: (a) present; **(b) absent**.
s7 Carpometacarpus, corpus carpometacarpi, os metacarpale majus, facies dorsalis, impressio m. extensor metacarpi ulnaris, position relative to synostosis metacarpalis proximalis: (a) completely proximal; **(b) opposite, at least in part**.
s13 Tarsometatarsus, extremitas distalis tarsometatarsi, canalis interosseus tendineus, osseus lamina covering dorsal (of two) canaliculi: (a) present; **(b) largely or completely lacking**.
t1 Syrinx, bulla syringealis (primary chamber): (a) solid; **(b) with fenestrae**.
t4 Syrinx, bulla syringealis, solid, bulbous, basal chamber: (a) absent; **(b) present**.
d13 Hallux, cutaneous lobation: (a) absent; **(b) present (small in *caryophyllacea*)**.
d19(a>b) Foot, color (adults, both sexes): (a) greenish or yellowish; (b) orange or reddish; **(c) gray**.
d24(a>b) Iris, color: (a) dark brown; (b) red (brighter in adults, males); (c) orange; (d) yellow.
d27 Rump, and usually also undertail coverts, contrasting blackish color: absent; **(b) present (vestigial to obsolete in *australis*)**.
d60 Proximal secondary remiges, fine black external margin: (a) absent; **(b) present**.

c) Monophyly *Netta* and *Aythya*

s14 Tarsometatarsus, corpus tarsometatarsi, relative dorsal prominence of facies subcutanea medialis and facies subcutanea lateralis: (a) equal, no torsion of corpus evident; **(b) medialis less prominent than lateralis, related to significant torsion of corpus about long axis**.
t2 2. Syrinx, bulla syringealis (primary chamber): (a) rounded; **(b) flattened "whorl"**.
d61 Secondary remiges, dark (sub)terminal band: (a) absent; **(b) present**.

d) Monophyly of *Aythya*

s4 Humerus, extremitas proximaIllihumeri, fossa pneumotricipitalis, foramen pneumaticum: (a) present; **(b) absent**.

s9 Femur, corpus femoris, craniocaudal curvature in lateral perspective: absent; **(b) present**.
s11 Tibiotarsus, extremitas distalis tibiotarsi, condyla medialis et lateralis, relative cranial prominence: (a) condylus medialis distinctly greater than condylus lateralis; **(b) condyla equally prominent**.
s12 Tibiotarsus, extremitas proximalis tibiotarsi, crista cnemialis cranialis, distinct ridge continuing distally along corpus tibiotarsi, facies cranialis, margo cranialis: (a) absent; **(b) present**.
d24(b>c,d), Iris, color: (a) dark brown; (b) red (brighter in adults, males); (c) orange; **(d) yellow**.

Supplementary Fig. S1. The maximum-likelihood tree from the analysis of a data set consisting of 2086 bp (cytochrome *b* and NADH dehydrogenase subunit 2 genes) obtained from 66 species of ducks, geese and swans. Numbers at the nodes are bootstrap values (after 100 replicates).

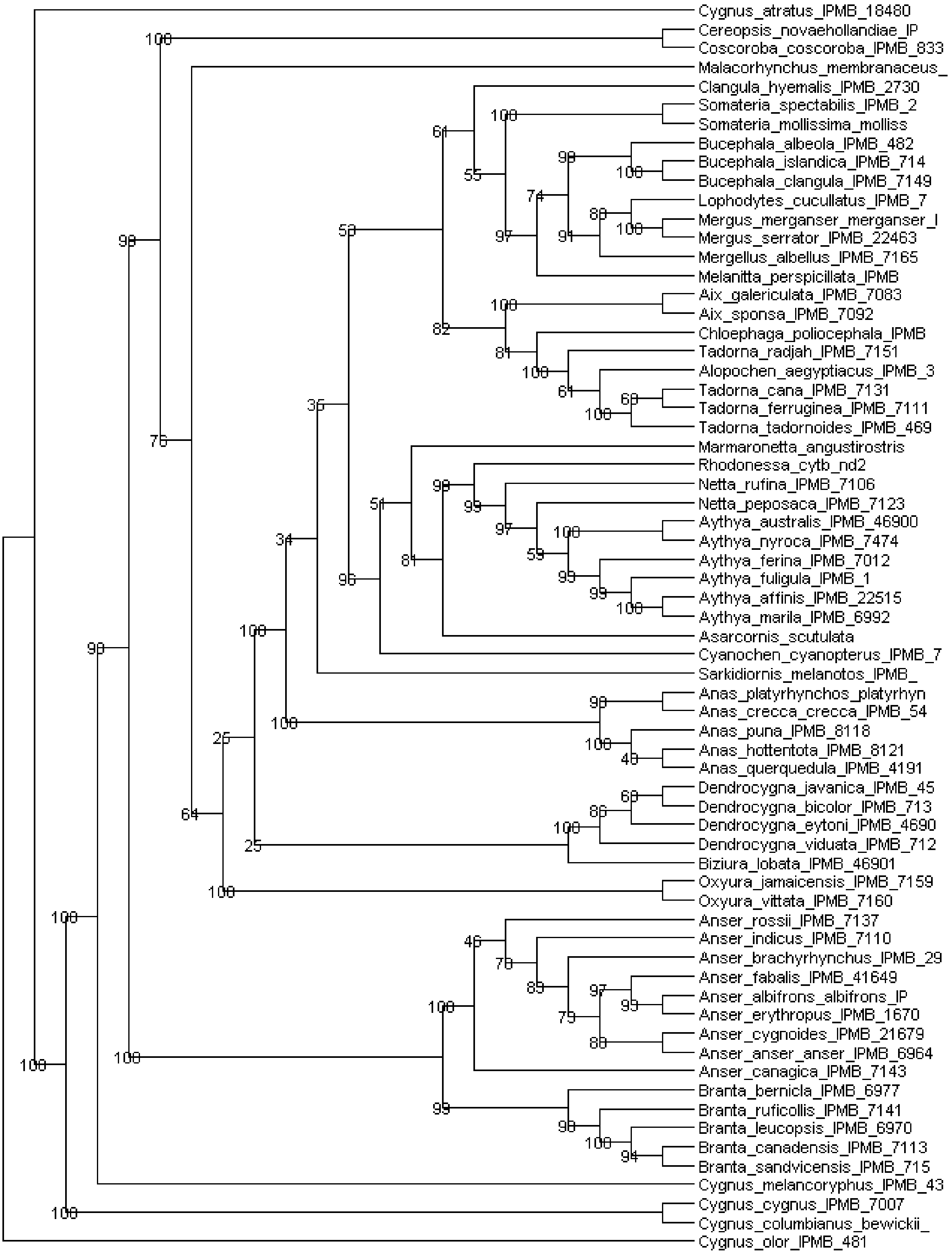

Supplement: Supplementary file 1 — Supplementary Information [file 41598_2017_16975_MOESM1_ESM.pdf]
